# Aberrant expression of B7-H4 and B7-H5 contributes to the development of cutaneous squamous cell carcinoma

**DOI:** 10.1007/s00403-024-03095-w

**Published:** 2024-06-08

**Authors:** Lu Chen, Bin Zhou, Minhui Tang, Yuxu Yao, Yue Zhao, Ying Hu, Yuxin Lin, Jiang Ji, Qingqing Jiao

**Affiliations:** 1 Department of Dermatology, The First Affiliated Hospital of Soochow University, Shizi Road 188, Suzhou, 215006 China; 2 Department of Dermatology, The Second Affiliated Hospital of Soochow University, Sangxiang Road 1055, Suzhou, 215004 China; 3 Jiangsu Institute of Clinical Immunology and Jiangsu Key Laboratory of Clinical Immunology, The First Affiliated Hospital of Soochow University, Suzhou, 215006 China; 4 Center for Systems Biology, Department of Bioinformatics, School of Biology and Basic Medical Sciences, Soochow University, Suzhou, 215123 China

**Keywords:** B7-H4, B7-H5, Cutaneous squamous cell carcinoma, Squamous cell carcinoma, Bioinformatical analysis, Tumor microenvironment

## Abstract

Cutaneous squamous cell carcinoma (CSCC) is the second most common malignant tumor of the skin. B7 homolog 4 (B7-H4) and B7-H5 (B7 homolog 5) are associated with a variety of tumors. Investigate the potential role of B7-H4 and B7-H5 in regulating the tumorigenesis and progression of CSCC. B7-H4 and B7-H5 transcriptome data were collected from GEO and TCGA databases and subjected to bioinformatical analysis by protein–protein interaction (PPI) network, functional enrichment analysis, immune analysis, and drug–gene interaction prediction analysis. We characterized the expression of B7-H4 and B7-H5 in carcinoma tissues of CSCC patients by immunohistochemistry. Meanwhile, the clinical correlation of B7-H4 and B7-H5 in CSCC was explored by statistical analysis. B7-H4 and B7-H5 genes were under-expressed in CSCC and correlated with tumor staging. According to GO and KEGG Pathway enrichment analysis, B7-H4, and B7-H5 can regulate the proliferation and activation of T cells, lymphocytes, and monocytes, and the expression of cytokines, such as IL-6 and IL-10, in CSCC. B7-H4 and B7-H5 are also jointly involved in the occurrence and development of CSCC via the JAK-STAT and Notch signaling pathways. We found that B7-H4 and B7-H5 proteins were abnormally highly expressed in CSCC tissue and correlated with tumor size and stage. Our findings offer new insights into the pathogenesis of CSCC and suggest that B7-H4 and B7-H5 are novel tissue biomarkers and promising therapeutic targets for CSCC.

**Supplementary Information:**

The online version contains supplementary material available at 10.1007/s00403-024-03095-w.

## Introduction

Cutaneous squamous cell carcinoma (CSCC) is the second most common malignant tumor of the skin after basal cell carcinoma, which is significantly affected by immunosuppression and immune dysregulation [1, 2]. Most patients with CSCC have a good prognosis by excision or local radiotherapy, but the prognosis for invasive phenotypes, local recurrence, or distant metastases is not ideal [3, 4]. Therefore, novel biomarkers and therapeutic targets are required for assessing risk and improving prognoses. Increasing evidence indicates that B7-H4 and B7-H5 are associated with the occurrence and development of a variety of tumors.

B7-H4 (B7 homolog 4), a V-set domain containing T-cell activation inhibitor-1 (VTCN1), belongs to the immune checkpoints of the immunoglobulin superfamily. Recent studies have demonstrated that B7-H4 is highly expressed in various tumor cells and tissues, and B7-H4 expression is associated with various pathological and clinical features [5, 6]. Studies have shown that B7-H4 is closely related to classical signaling pathways, such as IL-6/JAK/STAT3, PI3K/AKT, and CXCL12/CXCR4 [7]. Some B7-H4-based immunotherapy agents have been developed and achieved certain curative efficacy [8, 9].

B7-H5 (B7 homolog 5), a V-Set Immunoregulatory Receptor (VSIR), is also a member of the B7 immunoglobulin superfamily. Previous studies have found that B7-H5 is widely involved in various physiological and pathological processes, including regulating peripheral tolerance, inducing T-cell activation and differentiation, and mediating tumor immunity [10, 11]. Additionally, many clinical trials about B7-H5-targeted cancer therapy have been conducted or are in progress [12].

The function of B7-H4 and B7-H5 in immune regulation is complex and controversial [13, 14]. So far, no studies of B7-H4 and B7-H5 in CSCC exist. Therefore, this study is the first to investigate the expression of B7-H4 and B7-H5 in patients with CSCC and their roles in squamous cell carcinoma (SCC). We hope to provide new ideas and breakthrough points for the mechanism and treatment of CSCC and other SCC.

## Materials and methods

### Microarray data analysis

Two microarray datasets (GSE45164 and GSE98767) were downloaded from GEO (http://www.ncbi.nlm.nih.gov/geo). GSE45164 and GSE98767 were downloaded from GPL571 Platforms and GPL10558 Platforms respectively. GSE45164 did not include the B7-H5 transcriptome data, while GSE98767 did not include the B7-H4 transcriptome data of healthy control. We comprehensively analyzed the transcriptome data of B7-H4 and B7-H5 in CSCC from databases GSE45164 and GSE98767.

The original datasets GSE45164 and GSE98767 were analyzed using the “affy” package. The construction of the protein–protein interaction (PPI) network was realized via STRING (https://string-db.org/). The limma package was applied to identify the differentially expressed genes (DEGs). Gene ontology (GO) and Kyoto Encyclopedia of Genes and Genomes (KEGG) Pathway enrichment analysis results were obtained from the KOBAS 3.0 database. Four miRNA prediction databases (miRDB, miRWalk, RNA22, and RNAinter) were used to predict B7-H4 and B7-H5 related microRNAs. Gene set enrichment analysis (GSEA) was performed on transcriptome sequencing data of CSCC. ESTIMATE algorithm is based on single sample Gene Set Enrichment Analysis and generates stromal and immune scores. The correlation between B7-H4 and B7-H5 expression and Drug response was predicted by the CellMiner database (http://discover.nci.nih.gov/cellminer/).

We also obtained the RNA-seq data and corresponding clinical information about SCC from the TCGA dataset (https://portal.gdc.com/). The Kaplan–Meier (KM) analysis with log-rank test was used to calculate the overall survival (OS) of SCC patients. To assess the reliable results of immune score evaluation, we used immuneeconv which integrates six latest algorithms, including TIMER, xCell, MCP-counter, CIBERSORT, EPIC, and quanTlseq.

### Study conduct, patients, and control subjects

Ethical approval from the Ethics Committee of The First Affiliated Hospital of Soochow University was obtained prior to the study. After informed consent, this study collected 45 cases of surgically resected CSCC samples in Dermatology from January 2010 to April 2014. The control samples were six cases of normal skin tissues from plastic surgery operations. The patients ranged in age from 36 to 88 years (27 males, 18 females). The patients didn’t receive radiotherapy, chemotherapy, or immunotherapy before surgery. The diagnosis of CSCC was determined respectively by two diagnostic pathologists and two dermatologists.

### Immunohistochemical stain (IHC)

Sections were deparaffinized and then rehydrated in graded alcohols and water. Sections were then treated with 3% H_2_O_2_-methanol for 30 min and serum for 40 min. Sections were incubated with monoclonal antibodies against B7-H4 (ab252438, Abcam) or B7-H5 (ab257314, Abcam) overnight. After washing with PBS, sections were incubated with biotinylated secondary antibodies (SA00004-1, Proteintech) for 30 min, and incubated with streptavidin-conjugated peroxidase (ab7403, Abcam) for 30 min. Slides were exposed and counterstained with diaminobenzidine (SK4100, Vector Laboratories) and Mayer’s hematoxylin (ab128990, Abcam).

The IHC results were based on staining intensity and the positive cell percentage. There were four grades of staining strength: 0 score corresponds to no staining; 1 score corresponds to slightly yellow; 2 score corresponds to brownish yellow; 3 score corresponds to tan. There were five grades of the positive cell percentage (the average of five visual): 1 score: ≤ 25%, 2 score: 26–50%, 3 score: 51–75%, 4 score: > 75%. The final score was calculated by multiplying the grades of staining strength and the positive cell percentage. The score was determined as negative (−, score: 0–2), weakly positive (+, score: 3–4), moderately positive (++, score: 5–8), or strongly positive (+++, score: 9–12). Or the scores that exceeded 5 points were considered positive expressions [15]. The analysis for all samples was done in a blinded manner.

### Statistical analysis

Statistical analysis and figures were performed or made using GraphPad Prism 5 and R version 4.0.3. Significant differences between the groups were analyzed using a t-test, Mann–Whitney test, or Fisher’s exact test. The correlation was analyzed using Spearman’s or Pearson’s correlation coefficients. A P-value ≤ 0.05 was considered statistically significant.

## Results

### B7-H4 and B7-H5 genes were under-expressed in CSCC and correlated with disease staging

**Fig. 1:**
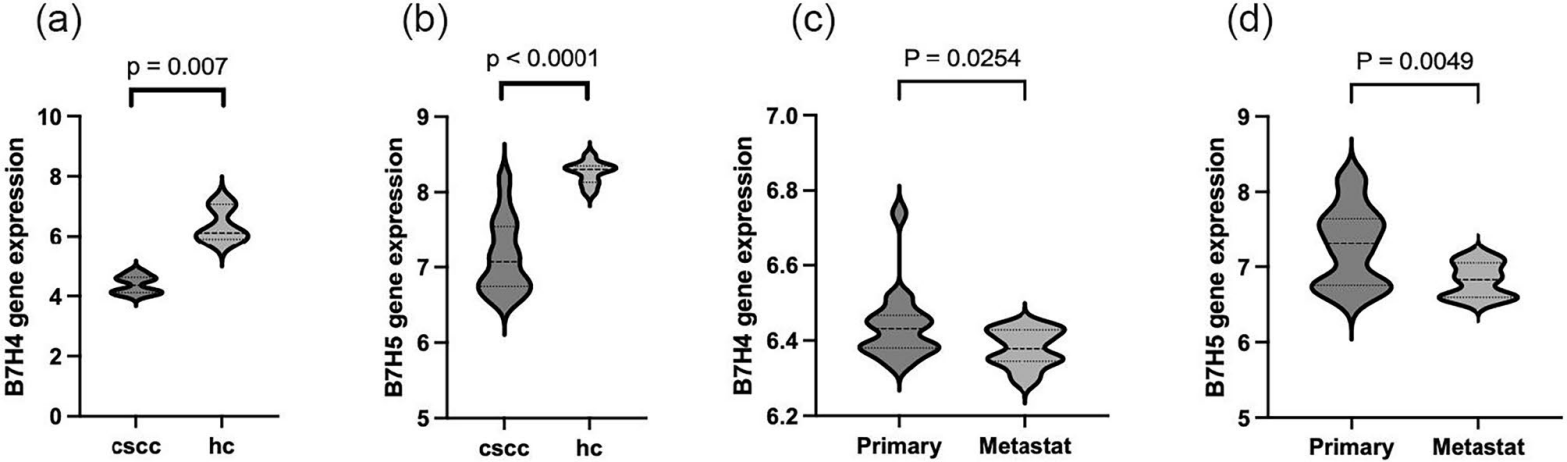
The gene expression levels of B7-H4 and B7-H5 in GSE45164 and GSE98767. B7-H4 (**a**) and B7-H5 (**b**) gene expression levels in CSCC and HC of GSE45164 and GSE98767. The gene expressions of B7-H4 (**c**) and B7-H5 (**d**) in different stages of CSCC from GSE98767

We identified differential expressions of B7-H4 and B7-H5 genes between the tumor sample and normal sample in GSE45164 and GSE98767. B7-H4 (p < 0.0001) and B7-H5 (p = 0.007) gene expressions in CSCC lesions were lower than that in normal skin tissue (Fig. 1a, b). Meanwhile, gene expressions of B7-H4 (p = 0.0254) and B7-H5 (p = 0.0049) in metastatic CSCC lesions were lower than that in primary CSCC lesions in GSE98767 (Fig. 1c, d).

### In CSCC, B7-H4 and B7-H5 were involved in the regulation of immune cells and related cytokines, and associated with the JAK-STAT and Notch pathways

**Fig. 2:**
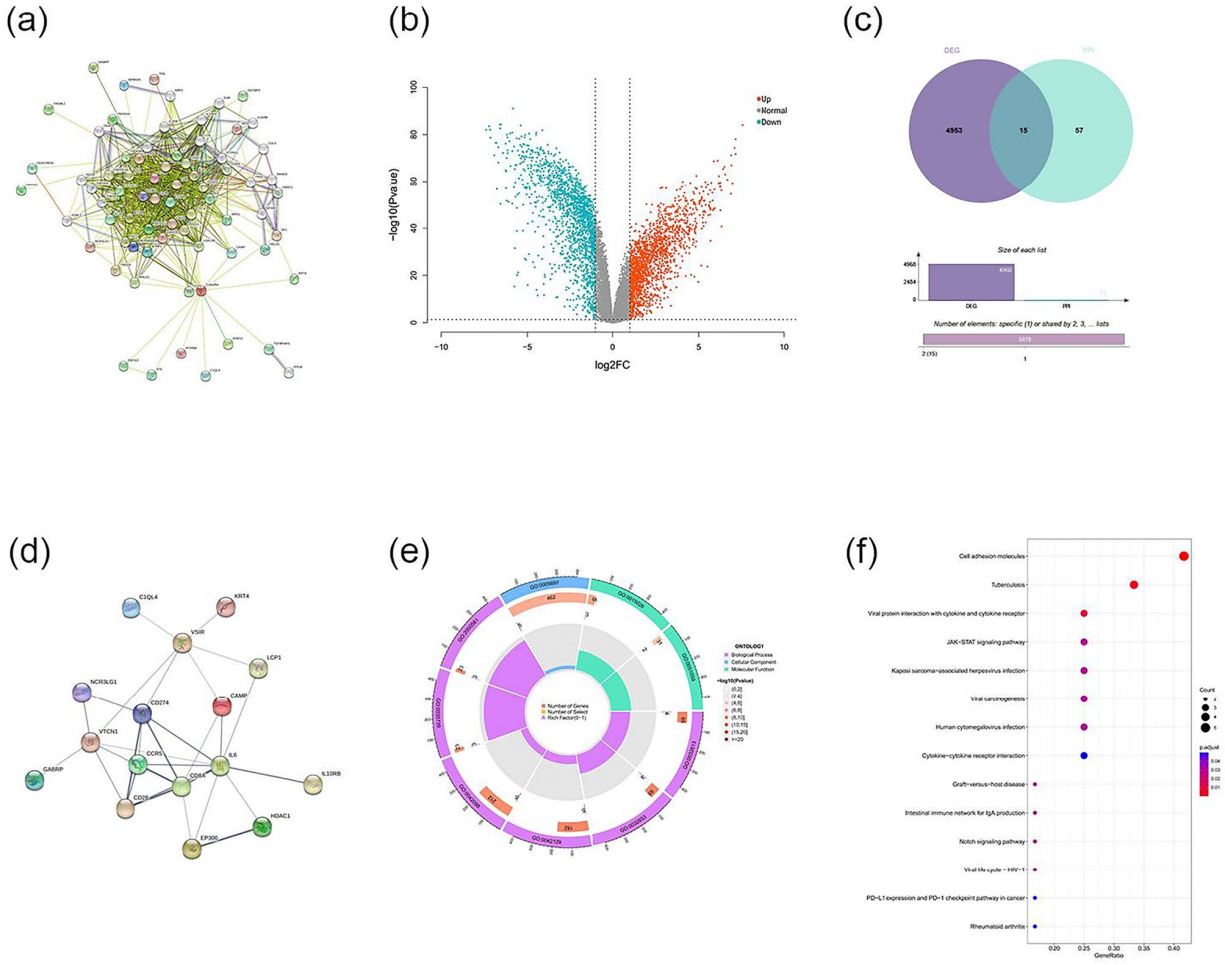
PPI network and DEGs enrichment analysis. **a** PPI network diagram. **b** The volcano map of GSE98767. **c** Overlap of 15 DEGs. **d** PPI network diagramof 15 DEGs and VTCN1. **e** The enrichment analysis results of GO pathway. **f** The enrichment analysis results of KEGG pathway. VTCN1 = B7H4, VSIR = B7H5

With B7-H4 and B7-H5 as the core, we screened 50 directly related proteins and 20 indirectly related proteins via STRING. Constructing a PPI protein network with a combined score greater than 0.4, 57 related genes were obtained (Fig. 2a). At the same time, we can also find that B7-H4 and B7-H5 were highly correlated at the protein level. After standardizing the microarray results, 4968 DEGs (in GSE98767) were identified (Fig. 2b). After taking the intersection of the Venn diagram, 15 hub genes were obtained (Fig. 2c). Adding B7-H4, we obtained a total of 16 hub genes related to B7-H4 and B7-H5 in CSCC, including C1QL4, KRT4, LCP1, CAMP, NCR3LG1, CD274, CCR5, CDBA, CD28, IL-6, IL-10RB, GABRP, HDAC1 and EP300 (Fig. 2d).

To analyze the biological functions and pathways of B7-H4 and B7-H5 in CSCC, GO and KEGG Pathway enrichment analyses were performed with the 16 hub genes. GO analysis results show that these genes were mainly enriched in positive regulation of T cell proliferation and activation, proliferation, and regulation of mononuclear and CD4-positive alpha–beta T cell, production and regulation of interleukin-10, external side of the plasma membrane, co-receptor activity, NF-κB binding (Fig. 2e). In terms of KEGG Pathway, the significant enrichment pathways are cell adhesion molecules, viral protein interaction with cytokine and cytokine receptor, JAK-STAT signaling pathway, viral carcinogenesis, cytokine-cytokine receptor interaction, Graft-versus-host disease, Notch signaling pathway, PD-L1 expression and PD-1 checkpoint pathway in cancer, etc. (Fig. 2f).

### MicroRNA prediction, pathway analysis, ESTIMATE algorithm, and Drug sensitivity analysis of B7-H4 and B7-H5

MicroRNA/mRNA interactions are a common mechanism for regulating gene expression. Based on four miRNA prediction databases, a total of 58 putative miRNAs of B7-H4 were collectively identified. And a total of 91 putative miRNAs of B7-H5 were collectively identified in those four databases (Figure S1a-b). At the same time, four miRNAs (hsa-miR-6885-5p, hsa-miR-328-5p, hsa-miR-125a-5p, and hsa-miR-4492) were screened to interact with B7-H4 and B7-H5 (Figure S1c).

According to the expression of B7-H4 and B7-H5, we divided them into high and low-expression groups for GSEA analysis. We showed that the first five significant pathways of B7-H4 were enriched: arginine biosynthesis, bladder cancer, phototransduction, prolactin signaling pathway, and proximal tubule bicarbonate reclamation (Figure S1d). And we also showed the first five significant enrichments of B7-H5, which were arachidonic acid metabolism, cytosolic DNA − sensing pathway, nitrogen metabolism, staphylococcus aureus infection and thyroid cancer (Figure S1e).

By using the ESTIMATE algorithm, we assessed immune or stromal components in TME. The estimate score between B7-H4 high expression group and the low expression group was significantly different (Figure S1f). And Immune Score in B7-H4 high expression group was high than that low expression group (Figure S1g).

We also analyzed the correlation between B7-H4 and B7-H5 expression and targeted therapy drug predictions. Multiple drugs were screened out and the first 16 results were visualized (Figure S2). It showed that B7-H4 was all sensitive to Acetalax, Bisacodyl, etc. B7-H5 was sensitive to Gefitinib, SW-044248, Erlotinib, etc. B7-H4 and B7-H5 were all resistant to XL-888, Ganetespib, Luminespib, etc. These analyses above can provide a reference for the selection of clinical therapeutic drugs.

### B7-H4 and B7-H5 expression levels were significantly higher in CSCC tissues

**Fig. 3:**
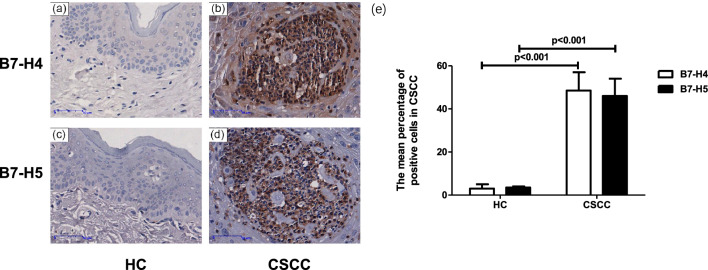
Representative immunohistochemically staining of expression of B7-H4 and B7-H5 molecule in different skin tumor tissues. B7-H4 (**a**) and B7-H5 (**c**) negative expression in normal skin tissues (n = 6). B7-H4 (**b**) and B7-H5 (**d**) positive expression in CSCC tissues (n = 45). (**e**) Expression of B7-H4 and B7-H5 in the tumor tissue from patients with CSCC. Magnification: 200 ×

As shown in Fig. 3, B7-H4 and B7-H5 were mainly distributed in the cell cytoplasm or membrane but less be found in nuclei. B7-H4 was observed in the tumor cells, immune cells, and stromal cells. B7-H5 was mostly expressed in immune cells and not in the tumor tissue. Moreover, B7-H4 was generally low detected in epidermis and sebaceous glands, but highly detected in eccrine glands. B7-H4 (Fig. 3a, b) and B7-H5 (Fig. 3c, d) expression in CSCC tissues was significantly higher than that in normal skin tissue (Fig. 3e). Of the total 45 CSCC cases, the percentage of B7-H4 high-expression patients was 57.78%, whereas the percentage of B7-H5 high-expression patients was 35.56%. In the same CSCC specimens, the expression of B7-H4 was higher than that of B7-H5.

### The correlation between B7-H4 and B7-H5 expression levels and clinic pathological variables of CSCC patients

We then analyzed the associations between B7-H4 and B7-H5 expression levels and age, gender, tumor size, UICC stage, or lymph-node metastasis. Among the 45 CSCC patients with potentially resected tumors, B7-H4 expression levels were significantly related to tumor size, and B7-H5 expression levels were significantly related to the UICC stage (Table 1). However, no association was observed between B7-H4 and B7-H5 expression levels and either age, gender, or lymph node metastasis.

**Table 1:** Correlation between B7-H4 and B7-H5 expression and clinicopathological variables of CSCC patients

Characteristics	Case	B7H4		P	B7H5		P
		Low	High		Low	High	
Gender							
Male	27	11	16	0.805	18	9	0.758
Female	18	8	10		11	7	
Age							
< 60 years	20	9	11	0.769	16	4	0.066
≥ 60 years	25	10	15		13	12	
Tumor size							
< 2 cm	22	17	5	< 0.001	14	8	0.912
≥ 2 cm	23	2	21		15	8	
UICC stage							
I/II	40	18	22	0.378	29	11	0.004
III/IV	5	1	4		0	5	
LN metastasis							
No	44	19	25	0.387	29	15	0.356
Yes	1	0	1		0	1	

### B7-H4 and B7-H5 mRNA expression levels, predictive value, and immune infiltration in SCC

**Fig. 4:**
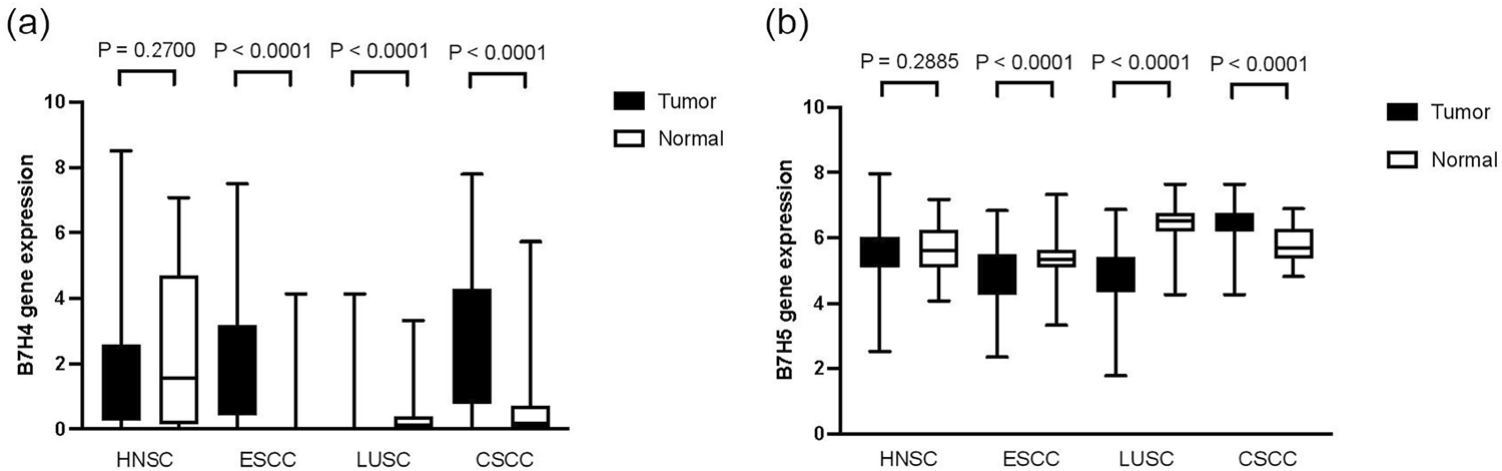
The gene expression levels of B7-H4 and B7-H5 in SCC. The gene expression levels of B7-H4 (**a**) and B7-H5 (**b**) in SCC and HC from TCGA dataset

To further define the role of B7-H4 and B7-H5 in SCC, we analyzed the mRNA expression, predictive value, and immune infiltration of B7-H4 and B7-H5 in other SCC using the TCGA dataset, such as HNSC, ESCC, LUSC, and CESC-CSCC. B7-H4 mRNA level is significantly upregulated in ESCC and CESC-CSCC and decreased in LUSC than normal samples (Fig. 4a). B7-H5 mRNA level is significantly upregulated in CESC-CSCC and decreased in ESCC and LUSC than in normal samples (Fig. 4b).

High B7-H4 mRNA levels showed a trend towards a good prognostic impact on OS but the difference failed to reach significance (Figure S3a, c–f). High mRNA expression levels of B7-H5 were associated with longer OS than low mRNA expression levels in CESC-CSCC (Figure S3b, j). However, High mRNA expression levels of B7-H5 were related to shorter OS in LUSC (Figure S3b, i).

We also explored the immune features of B7-H4 and B7-H5 in the tumor microenvironment among these SCC. B7-H4 mRNA expression levels were negatively related to most infiltrated immune cells in SCC (Figure S4a, c, e). B7-H5 mRNA expression levels were positively related to most infiltrated immune cells in SCC (Figure S4b, d, f).

## Discussion

Firstly, this study investigated B7-H4 and B7-H5 gene expressions, immunoregulation, signaling pathways, MicroRNA Prediction and therapy drug predictions in patients with CSCC through microarray analysis. Furthermore, we found that B7-H4 and B7-H5 proteins were abnormally highly expressed in tumor tissues and correlated with tumor size and stage in CSCC. Finally, we explored differences in gene expression levels of B7-H4 and B7-H5 in different SCC, which were correlated with overall survival and infiltrated immune cells in SCC.

Increasing numbers of research have uncovered that B7-H4 and B7-H5 were abnormally expressed in a variety of human malignant tumor tissues and correlated with disease prognosis [12, 16, 17]. In this study, it was found that B7-H4 and B7-H5 gene expression levels were down-regulated in keratinocytes cell lines of CSCC, and correlated with tumor staging. B7-H4 and B7-H5 can regulate the proliferation and activation of immune cells and the expression of cytokines. B7-H4 and B7-H5 were also jointly involved in the JAK-STAT and Notch signaling pathways. B7-H4 expression is increased and associated with IL-6 and p-STAT3 expression during SCC formation [18]. In the microenvironment of cervical cancer, expression levels of B7-H3 and B7-H4 positively correlated with the expression levels of IL-10 and TGF-β1, suggesting correlation with the p-JAK2/STAT3 pathway [19]. Furthermore, NOTCH1 has drawn much attention as it was identified as one of the most frequently mutated genes in the SCC of cutaneous, head and neck, esophageal, and lungs [20, 21]. Similar results were also found in many types of cancers, including renal cell carcinoma, ovarian cancer, and lung cancer [22, 23].

For resistance or non-response to existing targets, discovering new targets for treatment and developing alternative immunoregulatory therapies has been desperately demanded. The advantage of targeting B7-H4 and B7-H5 may be twofold, as they are expressed in tumor cells and tumor-associated immune cells of various cancer types. In this study, we found that the B7-H5 gene expression level was associated with drug sensitivity to Gefitinib. EGFR is widely expressed and mutated in NSCLC, which participates in immune evasion, most likely by regulating the expression of B7-H5 [24]. Clinically, Gefitinib is often used to treat these patients. Previous studies have revealed that B7-H4 is an antibody–drug conjugate (ADC) target for breast cancer and supports the possible use in the treatment of B7-H4^+^ breast cancer [25]. Furthermore, the signaling pathways of B7-H5 appear to be non-overlapping with CTLA-4 and PD-1/PD-L1 pathways. Combined blocking of immune checkpoint inhibitors may elicit synergistic effects in terms of stimulating anti-tumoral immune responses [26, 27].

B7-H4 and B7-H5 are commonly recognized to play potential roles in tumorigenesis and progression, autoimmune disease, and inflammatory responses. In this study, it was found that B7-H4 and B7-H5 proteins were highly expressed in CSCC tissue and correlated with tumor size and stage in CSCC. Interestingly, this was inconsistent with the gene expression results. It may also suggest that the translation of B7-H4 and B7-H5 in tumor tissues is regulated by other upstream and downstream genes. At the same time, there may be some differences in the tumor microenvironment between actual tumor tissue and cultured cell lines. Our results suggested that B7-H4 and B7-H5 may play an important role in the occurrence and development of CSCC. The complexity of the tumor microenvironment may significantly affect the expression of B7-H4 and B7-H5, and the different molecular mechanisms of this regulation need to be further clarified.

We also found that B7-H4 and B7-H5 were abnormally expressed in other SCC and correlated with disease prognoses, such as HNSC, ESCC, LUSC, and CESC-CSCC. However, B7-H4 and B7-H5 may have different functions in different SCC, suggesting that they may transcribe in a tissue-specific manner. This is consistent with previous research [5, 14, 28]. In oral SCC, B7-H5 overexpression has been associated with lymph node metastasis and the inferior overall survival of affected patients [29]. Upregulation of B7-H4 in OSCC relative to the control was also noted [30]. Previous studies have demonstrated that B7-H4 was highly expressed in human ESCC and linked to prognostic factors leading to poor clinical outcomes [31, 32]. In cervical cancer, B7-H4 and B7-H5 were more frequent in primary tumors than in recurrent counterparts and correlated with improved survival [33].

The tumor microenvironment (TME) influences tumor growth, metastatic spread, and response to treatment, comprising tumor cells, a vast variety of immune cells, and non-immune stromal cells [34]. In this study, the expression levels of B7-H4 and B7-H5 were associated with stromal and immune scores in CSCC. And we found a significant association between B7-H4 and B7-H5 expression levels and immune infiltration cells in SCC. B7-H4 may inhibit the expression and activation of CD8+ T cells, NK cells, M1 macrophages, monocytes, and Tregs in SCC. B7-H5 may promote the expression and activation of CD4+/CD8+ T cells, NK cells, M1 macrophages, monocytes, Tregs, and B cells in SCC. Zhou et al. have revealed that B7-H4 expression in tumor cells of mouse models negatively regulated CD8 tumor-specific T cell cytotoxicity, expansion, and activation [35]. Knockdown of B7-H4 may promote M1 macrophage polarization and inhibit M2 macrophage polarization via deactivating the PD-1/STAT3 pathway, thus restraining OSCC development [36]. Moreover, our results suggest that B7-H5 plays a key regulating factor in reprogramming and restraining macrophage inflammatory differentiation. Consistent with this, anti-VSIR antibody treatment was found to induce mediators involved in both M2 macrophage polarization and suppress mediators of M1 macrophage polarization [37].

In summary, we found that B7-H4 and B7-H5 gene expression levels were down-regulated in SCC keratinocytes cell lines of CSCC and correlated with tumor staging. In addition, GO and KEGG Pathway enrichment analysis revealed that B7-H4 and B7-H5 can regulate the proliferation and activation of T cells, lymphocytes, and monocytes, and the expression of cytokines, such as IL-6 and IL-10 in CSCC. B7-H4 and B7-H5 were also jointly involved in the occurrence and development of CSCC via the JAK-STAT and Notch signaling pathways. This study also found that B7-H4 and B7-H5 protein levels were abnormally high in tumor tissues and correlated with tumor size and stage in CSCC. B7-H4 and B7-H5 expression levels were also associated with the prognosis of multiple SCC and related to the regulation of immune infiltration cells in the tumor microenvironment in SCC. Therefore, B7-H4 and B7-H5 may be novel tissue biomarkers and promising therapeutic targets for CSCC.

## Supplementary Information

Below is the link to the electronic supplementary material.

Supplementary file1 Figure S1 MicroRNA Prediction, Pathway analysis and TME scores of B7-H4 and B7-H5. miRDB, miRWalk, RNA22, and RNAinter were used to predict the putative miRNAs targeting B7-H4 and B7-H5 (a-c). GSEA analysis for B7-H4 (c) and B7-H5 (d). ImmuneScore, StromalScore and ESTIMATEScore of B7-H4(e) and B7-H5 (f) mRNA expression. VTCN1=B7-H4, VSIR=B7-H5. Figure S2 Targeted therapy drugs prediction. VTCN1=B7-H4, C10orf54=B7-H5. Figure S3 Prognostic value of B7-H4 and B7-H5 in SCC. Survival analysis of B7-H4 (a) and B7-H5 (b) on OS in SCC described by the forest plot. K. M. analysis of B7-H4 in HNSC (c), ESCS (d), LUSC (e), CSCC (f). K. M. analysis of B7-H5 in HNSC (g), ESCS (h), LUSC (i), CSCC (j). Figure S4 Correlation between B7-H4 or B7-H5 and infiltrated inflammatory cells. Immune cell infiltration analyzed by the EPIC (a, b), MCPCOUNTER (c, d), CIBERSORT (e, f) algorithms. *p < 0.05, **p < 0.01, ***p < 0.001 (PDF 1270 KB)

## Data Availability

The data that supports the evidence presented in this article is accessible from public databases.

## Abbreviations

B7-H4: B7 homolog 4
B7-H5: B7 homolog 5
CSCC: Cutaneous squamous cell carcinoma
SCC: Squamous cell carcinoma
HNSC: Head and neck squamous cell carcinoma
ESCC: Esophageal squamous cell cancer
LUSC: Lung squamous cell cancer
CESC-CSCC: Cervical squamous cell carcinoma
GEO: Gene Expression Omnibus
TCGA: Gene set enrichment analysis
GSEA: Gene set enrichment analysis
DEGs: Differentially expressed genes
PPI: Protein–protein interaction
OS: Overall survival
TME: Tumor microenvironment
IL-6: Interleukin 6
IL-10: Interleukin 10
