# Supplementary figures and images for: Aberrant expression of B7-H4 and B7-H5 contributes to the development of cutaneous squamous cell carcinoma
Source: Arch Dermatol Res. 2024 Jun 8;316(7):382. doi: 10.1007/s00403-024-03095-w (PMC11162363; doi:10.1007/s00403-024-03095-w)

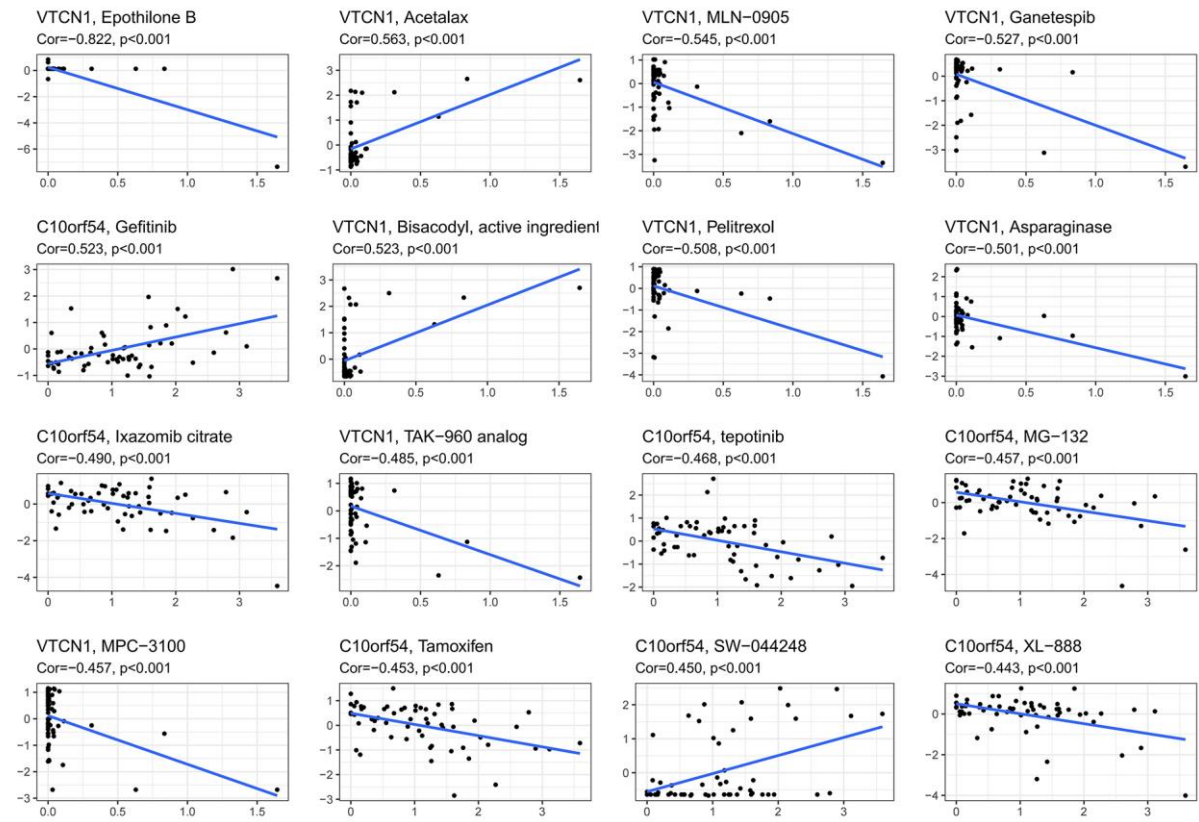

Figure S2

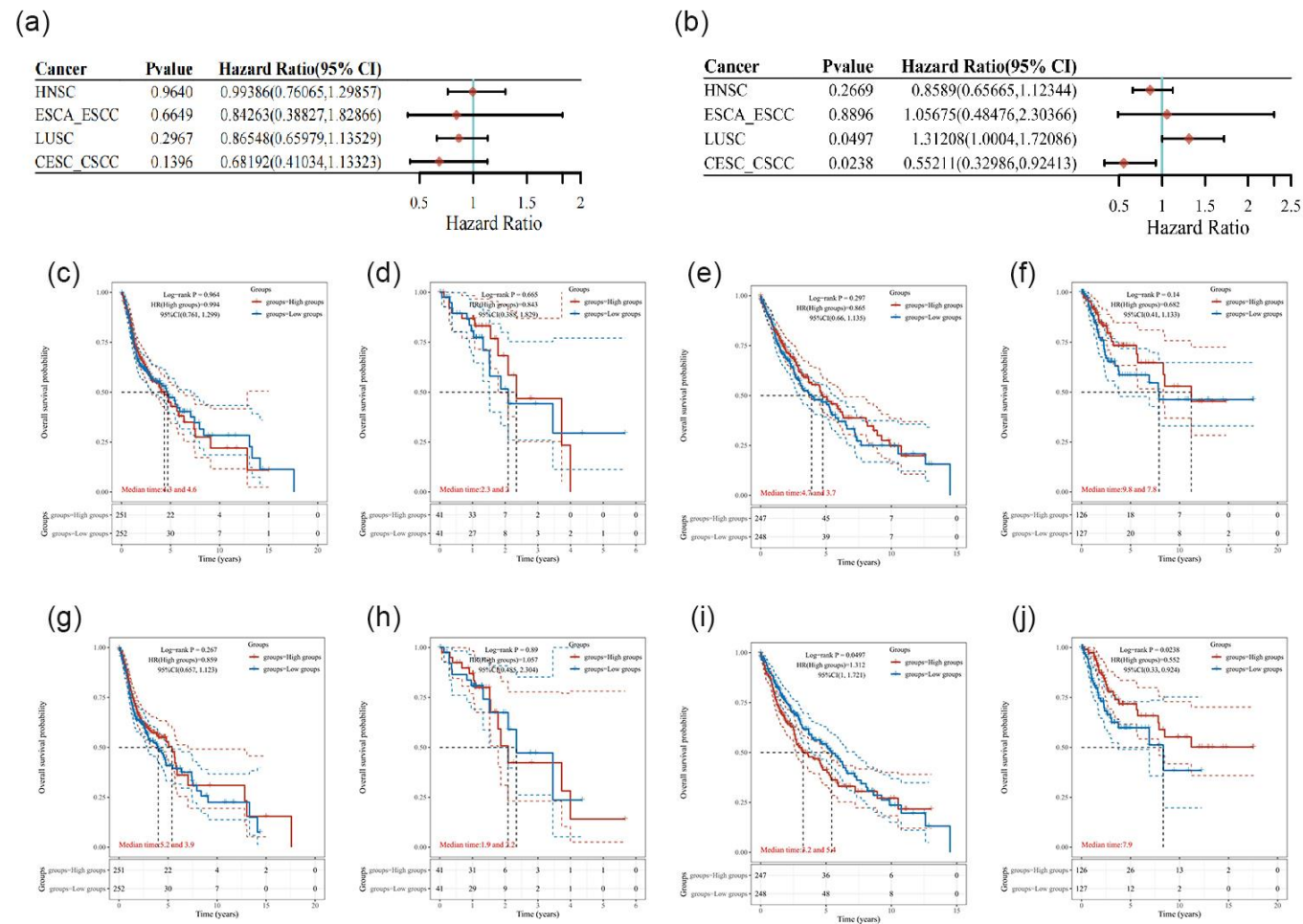

Figure S3

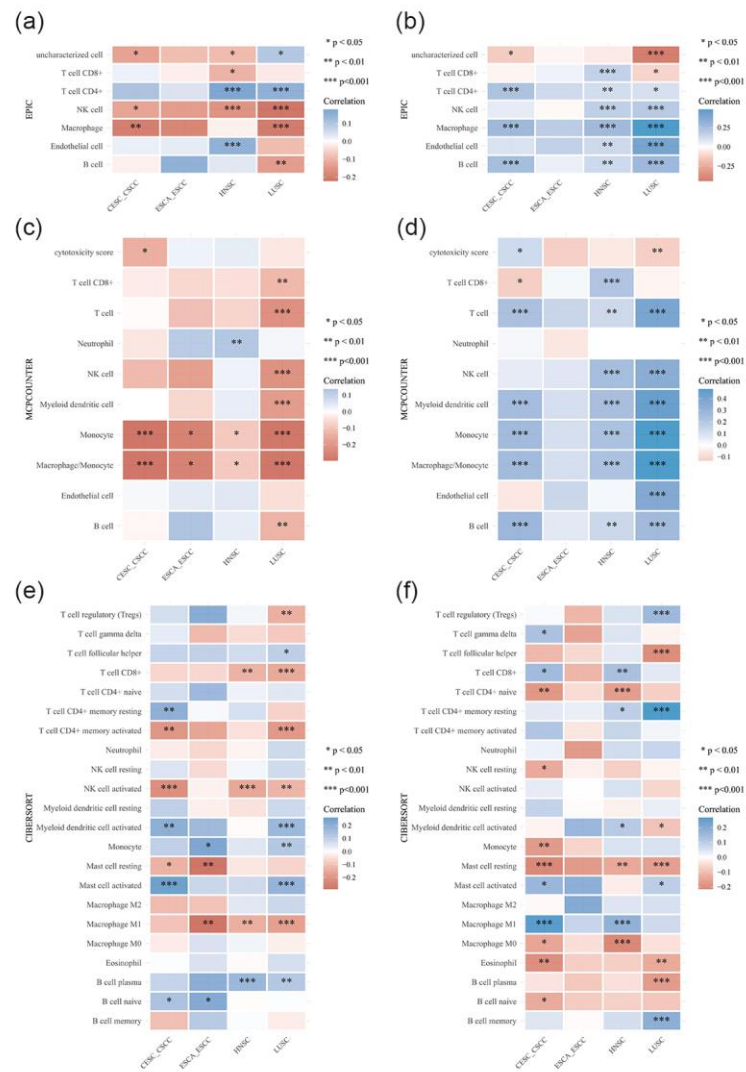

Figure S4

Supplement: Supplementary file 1 — Supplementary file1 Figure S1 MicroRNA Prediction, Pathway analysis and TME scores of B7-H4 and B7-H5. miRDB, miRWalk, RNA22, and RNAinter were used to predict the putative miRNAs targeting B7-H4 and B7-H5 (a-c). GSEA analysis for B7-H4 (c) and B7-H5 (d). ImmuneScore, StromalScore and ESTIMATEScore of B7-H4(e) and B7-H5 (f) mRNA expression. VTCN1=B7-H4, VSIR=B7-H5. Figure S2 Targeted therapy drugs prediction. VTCN1=B7-H4, C10orf54=B7-H5. Figure S3 Prognostic value of B7-H4 and B7-H5 in SCC. Survival analysis of B7-H4 (a) and B7-H5 (b) on OS in SCC described by the forest plot. K. M. analysis of B7-H4 in HNSC (c), ESCS (d), LUSC (e), CSCC (f). K. M. analysis of B7-H5 in HNSC (g), ESCS (h), LUSC (i), CSCC (j). Figure S4 Correlation between B7-H4 or B7-H5 and infiltrated inflammatory cells. Immune cell infiltration analyzed by the EPIC (a, b), MCPCOUNTER (c, d), CIBERSORT (e, f) algorithms. *p < 0.05, **p < 0.01, ***p < 0.001 (PDF 1270 KB) [file 403_2024_3095_MOESM1_ESM.pdf]
